# Next-generation sequencing facilitates quantitative analysis of wild-type and *Nrl^−/−^* retinal transcriptomes

**Published:** 2011-11-23

**Authors:** Matthew J. Brooks, Harsha K. Rajasimha, Jerome E. Roger, Anand Swaroop

**Affiliations:** Neurobiology-Neurodegeneration and Repair Laboratory, National Eye Institute, National Institutes of Health, Bethesda, MD

## Abstract

**Purpose:**

Next-generation sequencing (NGS) has revolutionized systems-based analysis of cellular pathways. The goals of this study are to compare NGS-derived retinal transcriptome profiling (RNA-seq) to microarray and quantitative reverse transcription polymerase chain reaction (qRT–PCR) methods and to evaluate protocols for optimal high-throughput data analysis.

**Methods:**

Retinal mRNA profiles of 21-day-old wild-type (WT) and neural retina leucine zipper knockout (*Nrl^−/−^*) mice were generated by deep sequencing, in triplicate, using Illumina GAIIx. The sequence reads that passed quality filters were analyzed at the transcript isoform level with two methods: Burrows–Wheeler Aligner (BWA) followed by ANOVA (ANOVA) and TopHat followed by Cufflinks. qRT–PCR validation was performed using TaqMan and SYBR Green assays.

**Results:**

Using an optimized data analysis workflow, we mapped about 30 million sequence reads per sample to the mouse genome (build mm9) and identified 16,014 transcripts in the retinas of WT and *Nrl^−/−^* mice with BWA workflow and 34,115 transcripts with TopHat workflow. RNA-seq data confirmed stable expression of 25 known housekeeping genes, and 12 of these were validated with qRT–PCR. RNA-seq data had a linear relationship with qRT–PCR for more than four orders of magnitude and a goodness of fit (R^2^) of 0.8798. Approximately 10% of the transcripts showed differential expression between the WT and *Nrl^−/−^* retina, with a fold change ≥1.5 and p value <0.05. Altered expression of 25 genes was confirmed with qRT–PCR, demonstrating the high degree of sensitivity of the RNA-seq method. Hierarchical clustering of differentially expressed genes uncovered several as yet uncharacterized genes that may contribute to retinal function. Data analysis with BWA and TopHat workflows revealed a significant overlap yet provided complementary insights in transcriptome profiling.

**Conclusions:**

Our study represents the first detailed analysis of retinal transcriptomes, with biologic replicates, generated by RNA-seq technology. The optimized data analysis workflows reported here should provide a framework for comparative investigations of expression profiles. Our results show that NGS offers a comprehensive and more accurate quantitative and qualitative evaluation of mRNA content within a cell or tissue. We conclude that RNA-seq based transcriptome characterization would expedite genetic network analyses and permit the dissection of complex biologic functions.

## Introduction

Next-generation sequencing (NGS) technology has launched a new era of enormous potential and applications in genomic and transcriptomic analyses [1-3]. With continued cost reductions and improved analytical methods, NGS has begun to have a direct impact on biomedical discovery and clinical outcome [4-6]. NGS has enabled “meta-genomic” studies to survey the genomes of organisms in a particular ecosystem [7], and decode the entire genomes of species ranging from bacteria [8,9] and viruses [10] to humans [11]. Whole-genome sequencing has made it possible to investigate genetic variations [12], global DNA methylation [13], and in vivo analysis of targets of DNA-binding proteins [14,15]. Deep sequencing of RNA with NGS (called “RNA-seq”) allows a comprehensive evaluation and quantification of all subtypes of RNA molecules expressed in a cell or tissue [16]. RNA-seq technology can detect transcripts expressed at low levels [17] and permit the identification of unannotated transcripts and new spliced isoforms [16,18]. The issues related to cross-hybridization and detection levels that limit the accuracy of gene expression estimates by microarray technology are not relevant to the data obtained with RNA-seq [19]. Visualization of mapped sequence reads spanning the splice junctions can also reveal novel splice forms of annotated genes in the mouse retina, which was not possible with earlier hybridization-based technologies. With a steady reduction in the costs of NGS, RNA-seq is now emerging as a method of choice for comprehensive transcriptome profiling.

The vertebrate retina exhibits a highly organized laminar structure that captures, integrates, and transmits visual information to the brain for further processing. Photoreceptors constitute more than 70% of the retinal cells and convert light into electrical signals [20]. Rod photoreceptors mediate dim light vision and can detect a single photon of light, while cone photoreceptors are responsible for daylight vision, color perception, and visual acuity [21,22]. Impairment of photoreceptor function leads to retinal degeneration with a more common pattern of rod death preceding the death of cones [23-25]. The neural retina leucine zipper (*Nrl*) gene encodes a basic-motif leucine zipper transcription factor necessary for determining rod photoreceptor cell fate and functional maintenance [26]. The *Nrl^−/−^* mouse, generated by creating a loss of function mutation in *Nrl*, has a cone-only retina that serves as a useful model for studies of cone biology [26-28].

Several previous investigations have elucidated the gene expression landscape specific to whole retina or retinal cell types and during development or aging. Serial analysis of gene expression [29-31] and cDNA eye gene arrays [32-36] were initially used to determine signatures of retinal gene expression. Oligonucleotide microarrays have since allowed a more comprehensive approach to expression profiling [37-41]. Microarray analyses of flow-sorted photoreceptors and single cells from dissociated retinas [42-44] have begun to reveal new insights into regulatory networks. Application of NGS technology greatly expands the power of expression profiling by identifying all transcripts and spliced isoforms in the tissue or cell type of interest.

Here, we have used the power of NGS-based RNA-seq analysis to investigate in depth the transcriptome of wild-type (WT) and *Nrl^−/−^* retinas and identified a set of differentially expressed genes and spliced isoforms. We have also taken advantage of the knowledge about *Nrl^−/−^* mice to optimize workflows for data analysis and compared our results with those obtained with microarray methods and quantitative reverse transcription polymerase chain reaction (qRT–PCR) analysis. Our studies illustrate that RNA-seq offers a more complete, accurate, and relatively faster approach for comparative and comprehensive analysis of retinal transcriptomes and for discovering novel transcribed sequences. Our validated data analysis workflow should also be beneficial for similar studies by other investigators. Raw data and workflow are available on the N-NRL/NEI website.

## Methods

### Animals and tissue collection

All investigations on mice were approved by the Animal Care and Use Committee of the National Eye Institute and followed the tenets of the Declaration of Helsinki. C57Bl/6J (referred to as wild type, WT) and *Nrl^−/−^* (on C57Bl/6J background [26]) mice were euthanized with CO_2_ inhalation. The retinas were excised rapidly, frozen on dry ice, and stored at −80 °C.

### RNA isolation

Fresh frozen mouse retinas were lysed with a mortar and pestle in TRIzol Reagent, and total RNA was isolated according to the manufacturer’s protocol (Invitrogen, Carlsbad, CA). RNA quality and quantity were assessed with the RNA 6000 Nano Kit (Agilent, Santa Clara, CA).

### NGS library construction

Whole retinal RNA samples were independently processed from three wild-type and three *Nrl^−/−^* mice at P21. Total RNA (1 μg) was used with the TruSeq mRNA-seq Sample Preparation Kit (Illumina, San Diego, CA) to construct cDNA libraries. The quality of the libraries was verified using the DNA-1000 Kit (Agilent) and quantitation performed with qRT–PCR using ABI 7900HT (Life Technologies, Carlsbad, CA), as suggested in the Sequencing Library qRT–PCR Quantification Guide (Illumina). Gene Expression Master Mix (Life Technologies) was used for the qRT–PCR reactions, and a titration of phiX control libraries was employed as the quantification standard.

### Illumina sequencing

Each cDNA library (10 pM) was independently loaded into one flow cell lane, and single-read cluster generation proceeded using the TruSeq SR Cluster Generation Kit v5 (Illumina). Sequencing-by-synthesis (SBS) of 70-nucleotide length was performed on a Genome Analyzer IIx running SCS2.8 software using SBS v4 reagents (Illumina). Base calling and chastity filtering were performed using RTA (real-time analysis with SCS2.8).

### Burrows–Wheeler transform-based short read aligner analysis workflow

Burrows–Wheeler Transform Aligner (BWA) [45] was used to align RNA-seq reads against the mouse reference genome (build mm9), downloaded and indexed from the University of California Santa Cruz (UCSC) genome browser database [46]. The resulting sequence alignment/map files were imported into Partek Genomics Suite (Partek Inc., St. Louis, MO) to compute raw and fragments per kilobase of exon model per million mapped (FPKM) reads normalized expression values of the transcript isoforms defined in the UCSC refFlat file. A stringent filtering criterion of FPKM value 1.0 (equivalent to one transcript per cell [16]) in at least one out of six samples was used to obtain expressed transcripts. The FPKM values of the filtered transcripts were log-transformed using log2 (FPKM+offset) with an offset=1.0. ANOVA (ANOVA) was then performed on the log-transformed data of the two groups (WT and *Nrl^−/−^*) to generate fold change and p values for each transcript. Y-chromosome transcripts were filtered out along with non-coding (nc) RNAs, mitochondrial DNA coded genes, pseudogenes, and predicted protein-coding genes. Differentially expressed mRNA isoforms were filtered for a fold change cutoff of 1.5 and p-value cutoff of 0.05. These criteria were implemented to enable a comparison with previous expression studies. Hierarchical clustering was performed using Cluster 3.0 software [47]. We used uncentered correlation as the distance metric. Heatmaps and dendrograms were generated using JavaTreeView software [48]. Aligned reads were visualized using the Integrated Genomics Viewer (IGV) [49].

### TopHat/Cufflinks-based analysis workflow

Raw reads that passed the chastity filter threshold were mapped using TopHat [50] to identify known and novel splice junctions and to generate read alignments for each sample. Genomic annotations were obtained from Ensembl in gene transfer format (GTF). Splice junctions from the six samples were combined into a master junctions file that was used as an input file for the second iteration of TopHat mapping. The transcript isoform level and gene level counts were calculated and FPKM normalized using Cufflinks. An FPKM filtering cutoff of 1.0 in at least one of the six samples was used to determine expressed transcripts. Differential transcript expression was then computed using Cuffdiff. The resulting lists of differentially expressed isoforms were filtered and sorted into the following categories: protein coding mRNA transcripts and ncRNA transcripts.

### qRT–PCR analysis

Reverse transcription (RT) reactions were performed using oligo(dT)20 with SuperScript II reagents (Life Technologies) according to the manufacturer’s protocol. cDNA synthesized from 2 μg of total RNA (1 μg for minus RT controls) was diluted to 100 μl (fivefold dilution), and from this 1 μl was used for each qRT–PCR reaction. The qRT–PCR reactions were performed in triplicate for TaqMan assays or in duplicate for the SYBR assays, using three biologic replicates per genotype, on a 7900HT Genetic Analyzer (Life Technologies). TaqMan assays were performed using TaqMan Gene Expression Master Mix and TaqMan Gene Expression Assays (Life Technologies) for genes listed in Table 1. The SYBR Green assays (Table 2) were performed using Power SYBR Green Master Mix (Life Technologies) and oligonucleotides at a final concentration of 200 nM. Oligonucleotides were designed using the Primer3 PCR Primer Design Tool [51] and synthesized by Integrated DNA Technologies (Coralville, IA). To eliminate complications due to contaminating genomic DNA in the RNA samples, qRT–PCR reactions were also performed with minus-RT control, using hypoxanthine guanine phosphoribosyl transferase (*Hprt*) primer pairs that can differentiate between mRNA and genomic DNA (data not shown). Differential expression analysis was performed using the ddCt method [52], with the geometric average of actin, beta (*ActB*) and *Hprt* as the endogenous controls [53].

**Table 1:** TaqMan assays employed for qRT–PCR validation

**TaqMan assay ID**	**Gene symbol**	**Gene name**
Mm00607939_s1	Actb	actin, b
Mm00504628_m1	Arr3	arrestin 3, retinal
Mm00437764_m1	B2m	b-2 microglobulin
Mm00474799_m1	Cadm3	cell adhesion molecule 3
Mm00432322_m1	Casp7	caspase 7
Mm00833234_m1	Cnga1	cyclic nucleotide gated channel a 1
Mm00489232_m1	Cngb3	cyclic nucleotide gated channel b 3
Mm00656724_m1	Egr1	early growth response 1
Mm00442411_m1	Esrrb	estrogen related receptor, b
Mm00438796_m1	Eya1	eyes absent 1 homolog (Drosophila)
Mm00445225_m1	Fabp7	fatty acid binding protein 7, brain
Mm99999915_g1	Gapdh	glyceraldehyde-3-phosphate dehydrogenase
Mm00492388_g1	Gnat1	guanine nucleotide binding protein, a transducing 1
Mm00492394_m1	Gnat2	guanine nucleotide binding protein, a transducing 2
Mm01197698_m1	Gusb	glucuronidase, b
Mm01318747_g1	Hprt1	hypoxanthine guanine phosphoribosyl transferase 1
Mm00833431_g1	Hsp90ab1	heat shock protein 90 kDa a, class B member 1
Mm01340839_m1	Mef2c	myocyte enhancer factor 2C
Mm00443299_m1	Nr2e3	nuclear receptor subfamily 2, group E, member 3
Mm00476550_m1	Nrl	neural retina leucine zipper gene
Mm00524018_m1	Nxnl1	nucleoredoxin-like 1
Mm00433560_m1	Opn1mw	opsin 1 (cone pigments), medium-wave-sensitive
Mm00432058_m1	Opn1sw	opsin 1 (cone pigments), short-wave-sensitive
Mm00476679_m1	Pde6b	phosphodiesterase 6B, cGMP, rod receptor, b
Mm00473920_m1	Pde6c	phosphodiesterase 6C, cGMP specific, cone, a prime
Mm01225301_m1	Pgk1	phosphoglycerate kinase 1
Mm00519814_m1	Reep6	receptor accessory protein 6
Mm00520345_m1	Rho	Rhodopsin
Mm00524993_m1	Rorb	RAR-related orphan receptor b
Mm01612986_gH	Rpl13a	ribosomal protein L13A
Mm02601831_g1	Rps26	ribosomal protein S26
Mm00774693_g1	Sall3	sal-like 3 (Drosophila)
Mm01249143_g1	Socs3	suppressor of cytokine signaling 3
Mm01277045_m1	Tbp	TATA box binding protein
Mm00726185_s1	Tubb4	tubulin, b 4
Mm01198158_m1	Ubc	ubiquitin C
Mm00457574_m1	Wisp1	WNT1 inducible signaling pathway protein 1

**Table 2:** SYBR Green assays employed for qRT–PCR validation

**Gene symbol**	**Gene name**	**Forward**	**Reverse**
Abca13 (Exon 53/55)	ATP-binding cassette, sub-family A (ABC1), member 13	GACCTTCTGAGATGGCCAAG	TTAACTCCAAGGAGCCCAAA
Abca13 (Exon 58/60)	ATP-binding cassette, sub-family A (ABC1), member 13	CGGTACCTCTGGCAAACAAT	GGAAATGGAGCTTCAAGCAG
Acoxl	acyl-CoA oxidase-like	TGCTGTATGGAACGAAGCTG	TGTGGAATGTTGAAGGCAGA
Akt3	thymoma viral proto-oncogene 3	CATCTGAAACAGACACCCGATA	GTCCGCTTGCAGAGTAGGAG
Cadm3	cell adhesion molecule 3	AGGGATTGTGGCTTTCATTG	CTAGGGGCTCAGGAGTTGTG
Ccdc24	coiled-coil domain containing 24	TGTCACATGTTGCAGAACGA	TCTAAGGCTGGGAATGGATG
Cd8a	CD8 antigen, alpha chain	GACATCTCAGCCCCAGAGAC	GCTTGCCTTCCTGTCTGACT
Cox5b	cytochrome c oxidase, subunit Vb	CGTCCATCAGCAACAAGAGA	ATAACACAGGGGCTCAGTGG
Ctss	cathepsin S	TAAAGGGCCTGTCTCTGTGG	GCCATCCGAATGTATCCTTG
Drd4	dopamine receptor D4	AGACTGCCCACCTCCCTTAC	AAGAAAGGCGTCCAACACAC
Dynlt3	dynein light chain Tctex-type 3	TTGATGGAGTTTTGGGTGGT	GGTACGGTTCTCCCATCTGA
Hr	hairless	GCCCTCTCTGCTCAGCTCTA	CGGACCACACCGTCTAAGTT
Klf9	Kruppel-like factor 9	ACAGTGGCTGTGGGAAAGTC	CATGCTTGGTGAGATGGTCA
Klhl3	kelch-like 3	GAGCACTGGGAGGAGCTATG	AGGAGGTTGGTCTGCTGAGA
Klhl33	kelch-like 33	AGCTTCTTCCCTTTGGTGGT	CTACAGCCACCGCTGACATA
Neurod1	neurogenic differentiation 1	GCGTTGCCTTAGCACTTCTT	AGGAGTGTGTGTTGGCATTT
Nipal1	NIPA-like domain containing 1	CCCACAAGAGGGAGAAGTCA	GTAAACAGGCTTCCGTTCCA
Pip5k1a	phosphatidylinositol-4-phosphate 5-kinase, type 1 alpha	GGGGAACACAGAGCACAAGT	GGTCTTCTGAGGCTCACTGC
Plekhf2	pleckstrin homology domain containing, family F (with FYVE domain) member 2	GTTGTCGGGTTCGACTGGA	TGCGTCTAGTATTCGCCTCAC
Rab18	RAB18, member RAS oncogene family	TGCACGCAAGCATTCTATGT	GGCTCTCTTCCCTGTGTGAC
Rgs22	regulator of G-protein signaling 22	GCCCAGAAGATCCTTGAACA	CGCCTTGTCCTCTTCTGTGT
Rpgrip1	retinitis pigmentosa GTPase regulator interacting protein 1	GCCATGCTACATGCTCAAGA	TTTGGATGGCCTGGTTTCTA
Sema7a	sema domain, immunoglobulin domain (Ig), and GPI membrane anchor, (semaphorin) 7A	TCTACAGCTCCCAACGATCA	GCTCACAGCTCTGTTCCACA
Txnip	thioredoxin interacting protein	TATGTACGCCCCTGAGTTCC	GTTCCCCGCTGTAGAGACTG
Wisp1	WNT1 inducible signaling pathway protein 1	GCTCTACCACCTGTGGCCTA	ACAGCCTGCGAGAGTGAAGT
Wscd2	WSC domain containing 2	TCTGCATCAAGACCCATGAA	ACGGTCTTGCCAAACTTGAG

## Results

### Sequencing run summary

**Figure 1:**
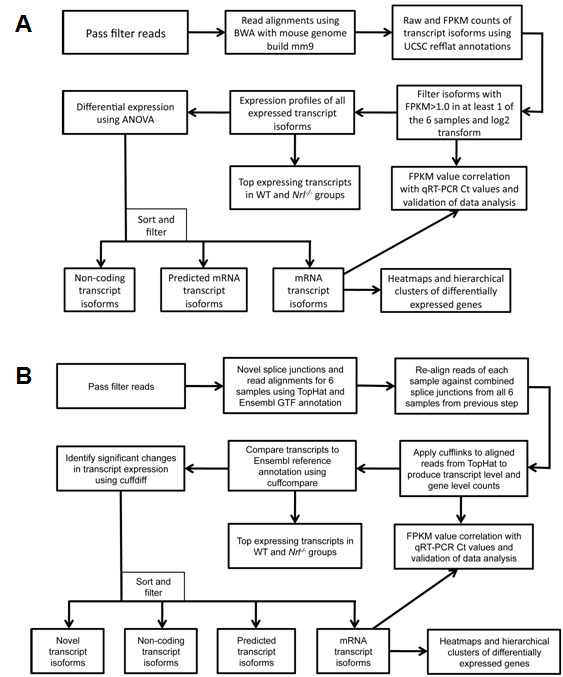
Flowchart of RNA-seq data analysis methodology using Burrows-Wheeler Aligner (BWA) and TopHat. Schematic representation of two RNA-seq data analysis workflows and resulting views of the data generated. **A**: BWA workflow: Gapped alignments are performed using the BWA algorithm against the mouse reference genome build mm9, and estimation of the expression of genes at the transcript isoform level is performed by importing aligned reads into the Partek Genomics Suite using annotations provided by the University of California Santa Cruz (UCSC) refflat.txt file. Transcripts expressed at low levels in all samples (<1 fragments per kilobase of exon model per million mapped reads [FPKM]) are filtered out. Differential expression analysis was performed by applying the ANOVA (ANOVA) method, and the resulting list was sorted and filtered into different transcript groups. Clustering of rod and cone enriched genes was performed using Cluster 3.0 software (see Methods). **B**: TopHat workflow: Splice junction mapping was performed using the TopHat algorithm in two phases. In the first phase, splice junctions were detected de novo from the reads from each sample and combined to obtain a master splice junctions list. In the second phase of TopHat alignment, reads from each sample were re-aligned by providing the master junctions list as input. The two-phase mapping approach significantly increased the number of alignments spanning the splice junctions. Estimation of gene expression and differential expression were computed using Cufflinks, Cuffcompare, and Cuffdiff. Sorting and filtering of transcript isoforms were performed as in the BWA workflow.

Six libraries of P21 retinal cDNA (three each from WT and *Nrl^−/−^*) were sequenced to obtain 35 to 49 million raw sequence reads per sample (Table 3). Of these, 75.8% to 82.7% reads passed the RTA chastity filter and were used for subsequent Burrows–Wheeler Aligner (BWA) and TopHat analysis workflows (Figure 1). Due to TopHat workflow’s power to map across splice junctions, the workflow consistently yielded 6 to 7 million more alignments per sample when compared to BWA.

**Table 3:** Summary of Illumina base calling and alignments

**Genotype**	**WT**	**WT**	**WT**	**Nrl^−/−^**	**Nrl^−/−^**	**Nrl^−/−^**
	**Sample 1**	**Sample 2**	**Sample 3**	**Sample 1**	**Sample 2**	**Sample 3**
Total reads	35,872,080	41,785,800	49,076,400	46,689,240	48,480,240	48,656,040
PF Reads	29,603,280	33,251,160	37,642,800	36,472,800	37,119,960	36,823,320
	82.7%	79.7%	76.9%	78.2%	76.7%	75.8%
BWA alignments	24,992,271	27,922,997	32,085,799	30,960,565	31,374,578	31,257,335
TopHat alignments	30,769,939	34,177,120	39,222,596	38,289,469	38,744,790	38,593,533

Each of the 3 week old WT and *Nrl^−/−^* retina sample was sequenced on a separate lane of the Illumina GAIIx flow cell to obtain 35 to 49 million raw reads. Over 75% of the raw reads passed Illumina’s read chastity threshold to yield 29 to 37 million usable PF reads. TopHat mapping always gave significantly more alignments than BWA because of its ability to map across the splice junctions. A relatively smaller numbers of reads and alignments for WT samples 1 and 2 are not a matter of concern as FPKM normalization was used to assess the transcript isoform expression. WT=wild type. PF=pass filter

### BWA workflow

Based on the BWA analysis workflow, 16,014 transcripts were detected with a normalized FPKM value greater than 1.0 in any of the six samples. Transcripts were filtered based on whether they were mRNAs or ncRNAs. Of the 15,142 mRNA transcripts, only 1,422 met our criteria of differential expression of having a fold change greater than 1.5 and a p-value less than 0.05 (Table 4). Of the 1,422 differentially expressed mRNA transcripts (DETs) representing 1,218 unique genes, 551 were downregulated in *Nrl^−/−^* (including rod-specific genes) retinas, and 871 were upregulated in *Nrl^−/−^* (including cone-enriched genes and those involved in retinal remodeling) retinas.

**Table 4:** Summary of transcript isoforms detected by BWA/ANOVA and TopHat/Cufflinks workflows

**Analysis**	**BWA/ANOVA**	**TopHat/Cufflinks**
Total detected transcripts	16,014	34,115
mRNA	15,142	32,001
mRNA DETs	1,422	3,258

The BWA workflow employed refflat.txt annotation for mouse build mm9 from UCSC genome browser. The TopHat workflow employed GTF annotation for mouse build mm9 from the Ensembl database. After FPKM filtering (see Materials and Methods), transcribed features were classified as protein coding mRNAs and non-coding (nc) RNAs. The features classified as protein-coding mRNAs were further filtered based on fold change (≥1.5) and p-value (<0.05) to be considered significantly differentially expressed transcripts (DETs).

### TopHat workflow

A total of 34,115 transcripts were detected with a normalized FPKM value of greater than 1.0 in any of the samples in either group. Transcripts were filtered based on whether they were protein-coding mRNAs or ncRNAs. Of the 32,001 mRNA transcripts, only 3,258 met our criteria of differential expression (Table 4). The DETs represented 1990 unique genes; 1,504 were downregulated in *Nrl^−/−^* (including rod-specific genes) retinas, and 1,754 were upregulated in the *Nrl^−/−^* (including cone-enriched genes and those involved in retinal remodeling) retinas.

### Comparison of the results from BWA and TopHat analyses

**Figure 2:**
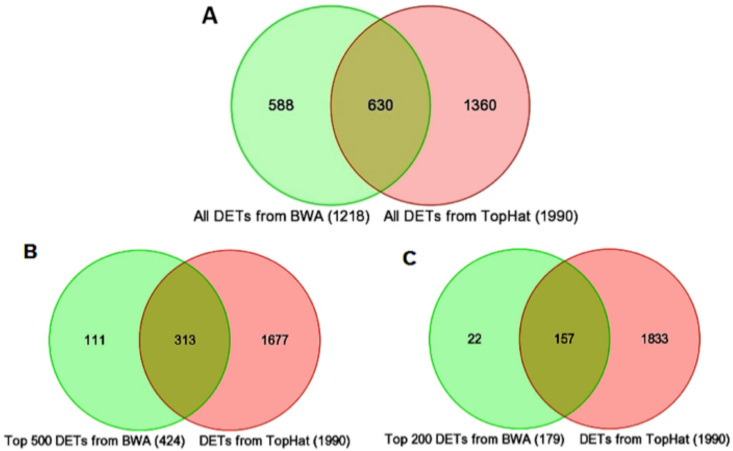
Venn diagrams comparing differentially expressed transcripts (DETs) between the *Nrl^−/−^* and WT groups from BWA and TopHat analyses. Despite major differences in the UCSC refFlat annotations used by Burrows-Wheeler Aligner (BWA) and Ensembl annotations used by TopHat, most of the genes identified by BWA were also identified as significant by TopHat. **A**: Comparison of the total number of DETs identified as significant (fold change ≥1.5 and p-value <0.05) by the two methods. **B**: Inclusion of the top 500 DETs (424 unique genes) identified as significant by BWA and in the full TopHat DET list. **C**: Inclusion of the top 200 DETs (179 unique genes) identified as significant by BWA and in the full TopHat DET list. We assess the two methods based on a comparison of qRT–PCR data for the genes detected by either or both methods. The discrepancy between the results can be attributed to differences in the input annotation files used (UCSC refFlat versus Ensembl GTF) by the two methods and their alignment algorithms.

The BWA/ANOVA and TopHat/Cufflinks analyses were compared to assess the consistency and quality of the results. Using the official Mouse Genome Informatics gene symbol as the linking term, Venn diagrams were constructed to summarize the overlap between the set of all (Figure 2A), the top 500 (Figure 2B), and the top 200 (Figure 2C) DETs from the BWA workflow and the DET list from the TopHat workflow. A comparison of the full list of BWA DETs to the TopHat list revealed only 51.7% overlap between the differentially expressed genes (DEGs) from BWA and TopHat. This overlap increased to 73.8% and 87.8% when only the top 500 and 200 DEGs from BWA, respectively, were considered. Subsequent analyses were performed using BWA data.

### Regression analysis of quantitative expression values obtained with RNA-seq and TaqMan qRT–PCR assays

**Figure 3:**
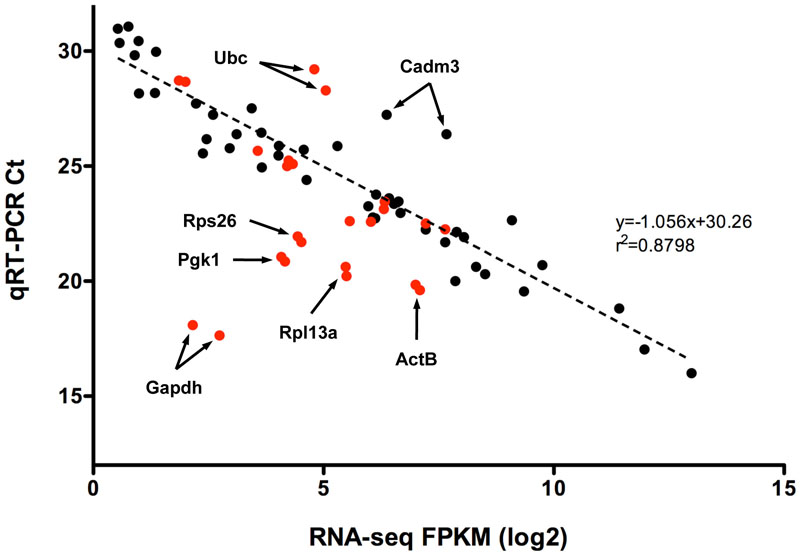
Correlation of RNA-seq and qRT–PCR. The correlations between the RNA-seq fragments per kilobase of exon model per million mapped reads (FPKM) values and the corresponding qRT–PCR crossing threshold (Ct) values are shown. FPKM values represented in log_2_ scale, and non-normalized Ct values are an average of three biologic replicates. Data generated from differentially expressed genes (black) is contrasted with data generated from the housekeeping genes (red). The dashed line, associated equation, and goodness of fit value were generated by least-squares regression analysis of the differentially expressed data set. Since a lower Ct value indicates an increased initial amount of target mRNA, an inverse relationship between FPKM and Ct values is expected if a correlation exists.

We first assessed the correlation between the FPKM values (obtained with RNA-seq) with their corresponding qRT–PCR crossing threshold (Ct) values from the TaqMan assays; the two values represent the quantitative levels of expression of a specific transcript in the RNA sample. For this purpose, we chose 24 differentially expressed genes (DEGs, reflecting a wide range of expression) and 12 housekeeping genes (HKGs). The Ct values from three biologic replicates (without normalization) were then compared to the corresponding log2 FPKM values (Figure 3). A least-squares regression analysis of DEGs provided an R^2^ value of 0.8798, with a corresponding slope of −1.056, suggesting a strong inverse relationship between Ct and log2 FPKM values over a dynamic range of 4–5 orders of magnitude. Only one out of 24 genes, cell adhesion molecule 3 (*Cadm3*), fell outside this correlation. Further investigation of the RNA-seq aligned reads showed that our qRT–PCR assay was specific for only one of the two retina-expressed spliced isoforms of *Cadm3*. The reanalysis using a SYBR assay designed to detect both *Cadm3* transcripts confirmed the linear correlation between RNA-seq and qRT–PCR analysis. Interestingly, FPKM and Ct values for 6 of the 12 HKGs did not show the expected linear relationship; these included ubiquitin C (*Ubc*), *ActB*, ribosomal protein L13A (*Rpl13a*), ribosomal protein S26 (*Rps26*), phosphoglycerate kinase 1 (*Pgkl*), and most severely glyceraldehyde-3-phosphate dehydrogenase (*Gapdh*). With the exception of *Ubc* that was underestimated by qRT–PCR (in the same manner as *Cadm3*), the BWA workflow underestimated all others.

### A comparison of RNA-seq and qRT–PCR data for housekeeping genes

RNA-seq data were evaluated for the expression of 27 established HKGs (Table 5) included in the control qRT–PCR plates from the following vendors: Life Technologies (Mouse Endogenous Control Array), SA Biosciences, Frederick, MD (Mouse Housekeeping Genes RT^2^ Profiler PCR Array), and Qiagen, Valencia, CA (QuantiTect Housekeeping Genes). Comparison of qRT–PCR data for 12 genes (that were tested) showed almost complete concordance of expression with the RNA-seq results. Only one gene, *Ubc*, revealed a significant difference in expression between the WT and *Nrl^−/−^* retinas with qRT–PCR (−1.89 fold) compared to the RNA-seq (−1.19 fold) analyses. *Gapdh* showed a relatively high change in expression in qRT–PCR and RNA-seq (−1.49 and −1.37 fold, respectively). *Hprt* and *Rpl13a* revealed lower variation in qRT–PCR and RNA-seq, respectively. *Actb*, TATA box binding protein (*Tbp*), glucuronidase, beta (*Gusb*), and *Pgk1* were among the best HKGs for qRT–PCR and RNA-seq normalization. For further qRT–PCR analyses, we employed *ActB* and *Hprt* in all normalization calculations.

**Table 5:** Quantitative expression profiles of housekeeping genes obtained by qRT–PCR and RNA-seq

**Transcript**	**Gene ID**	**Description**	**WT FPKM**	**Nrl^−/−^ FPKM**	**RNA-seq FC**	**WT Ct**	**Nrl^−/−^ Ct**	**qPCR FC**
NM_007393	Actb	Actin, b	136.24	128	−1.07	19.61	19.84	−1.17
NM_020559	Alas1	Aminolevulinic acid synthase 1	8.06	8.46	1.05			
NM_009735	B2m	β 2-microglobulin	**11.88**	**20.11**	**1.71**	**25.67**	**25.09**	**1.5**
NM_019468	G6pd2	Glucose-6-phosphate dehydrogenase 2	9.85	12.13	1.23			
NM_008084	Gapdh	Glyceraldehyde-3-phosphate dehydrogenase	**6.68**	**4.47**	**−1.49**	**17.64**	**18.09**	**−1.37**
NM_010368	Gusb	b-glucuronidase	3.63	4	1.1	28.72	28.67	1.03
NM_008194	Gyk	Glycerol kinase	2.62	2.69	1.02			
NM_001110251	Hmbs (isoform 2)	Hydroxymethylbilane synthase	3.16	3.25	1.03			
NM_013551	Hmbs (isoform 1)	Hydroxymethylbilane synthase	1.89	2.1	1.11			
NM_013556	Hprt	Hypoxanthine guanine phosphoribosyl transferase	47.5	65.34	1.37	22.61	22.58	1.02
NM_008302	Hsp90ab1	Heat shock protein 90 kDa α class B member 1	149.09	199.47	1.33	22.51	22.25	1.2
NM_001081113	Ipo8	Importin 8	10.48	11.31	1.08			
NM_023144	Nono	Non-POU-domain-containing octamer-binding	28.64	38.59	1.35			
NM_008828	Pgk1	Phosphoglycerate kinase 1	17.88	16.91	−1.06	20.86	21.05	−1.15
NM_009089	Polr2a	Polymerase (RNA) II (DNA directed) polypeptide A	50.56	36.5	−1.39			
NM_008907	Ppia	Peptidylprolyl isomerase A (cyclophilin A)	2.38	2.68	1.13			
NM_009438	Rpl13a	Ribosomal protein L13A	45.25	44.63	−1.01	20.22	20.62	−1.32
NM_007475	Rplp0	Ribosomal protein large P0	39.12	39.67	1.02			
NM_026020	Rplp2	Ribosomal protein large P2	18.51	17.15	−1.07			
NM_013765	Rps26	Ribosomal protein S26	22.94	21.71	−1.06	21.7	21.94	−1.19
NM_023281	Sdha	Succinate dehydrogenase complex subunit A, flavoprotein	74.03	80.45	1.09			
NM_013684	Tbp	TATA box binding protein	18.51	18.9	1.02	25	25.24	−1.18
NM_011638	Tfrc	Transferrin receptor	34.3	37.53	1.09			
NM_011654	Tuba2	Tubulin, α 2	37.53	46.85	1.25			
NM_009451	Tubb4	Tubulin β 4	79.34	80.45	1.01	23.13	23.45	−1.25
NM_019639	Ubc	Ubiquitin C	**33.13**	**27.86**	**−1.19**	**28.29**	**29.21**	**−1.89**
NM_011740	Ywhaz	Tyrosine 3-monooxygenase-tryptophan 5-monooxygenase activation protein z polypeptide	38.05	51.98	1.37			

We evaluated housekeeping genes (HKGs) for their expression in RNA-seq data and by qRT–PCR analysis. Rows in bold indicate genes that are significantly differentially expressed and hence not good choices for HKGs. WT=wild type, Ct=crossing threshold, FPKM=fragments per kilobase of exon model per million mapped reads.

### A comparison of RNA-seq and qRT–PCR analysis for DEGs

**Figure 4:**
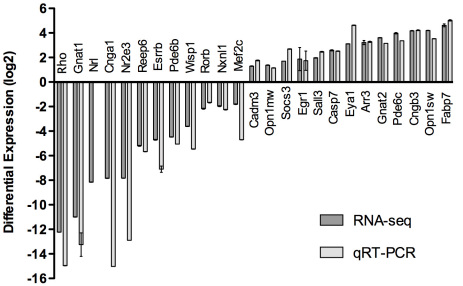
qRT–PCR validation of RNA-seq results. Comparison of differential expression values determined by RNA-seq (dark gray) and qRT–PCR (light gray) for 25 differentially expressed genes identified by Burrows-Wheeler Aligner (BWA) workflow. Error bars represent the standard error of the mean. Neural retina leucine zipper gene (*Nrl*) was not detectable by qRT–PCR and therefore are left blank in the graph. Note that Rhodopsin (*Rho*), guanine nucleotide binding protein, alpha transducing 1 (*Gnat1*), cyclic nucleotide gated channel alpha 1 (*Cnga1*), and nuclear receptor subfamily 2, group E, member 3 (*Nr2e3*) having average crossing threshold (Ct) values greater than 30 in the *Nrl^−/−^* samples are considered extremely low to non-expressing.

Based on the RNA-seq data from the WT and *Nrl^−/−^* retinas, we selected 25 DEGs (12 downregulated and 13 upregulated) showing a wide range of differential expression for validation with qRT–PCR analysis. qRT–PCR data for all genes validated the RNA-seq results (Figure 4). The WNT1 inducible signaling pathway protein 1 (*Wisp1*) TaqMan assay did not produce an amplicon in any of the experiments performed; subsequent examination of the RNA-seq data revealed that this assay did not correspond to the splice variant expressed in the retina. Additional analysis using a SYBR assay with oligonucleotides specific to the retinal splice variant confirmed the differential expression of *Wisp1* (−43.9 fold change) in the *Nrl^−/−^* retina compared to the WT.

### Expression levels of transcripts in the WT and *Nrl^−/−^* retina

The preceding analysis clearly demonstrates the high reliability and accuracy of the data obtained with RNA-seq methodology. We therefore used RNA-seq data to derive absolute expression levels of individual transcripts. The top 25 genes highly expressed in the WT or *Nrl^−/−^* retina are listed in Table 6 and Table 7. As predicted, most of these genes encode proteins involved in photoreceptor function/metabolism.

**Table 6:** Top 25 highly expressed transcripts in wild-type retina based on RNA-seq data.

**Transcript ID**	**Gene ID**	**Gene name**	**WT FPKM**	***Nrl^−/−^* FPKM**
NM_145383	*Rho*	Rhodopsin	8135.4	1.7
NM_008140	*Gnat1*	Guanine nucleotide binding protein γ transducing activity polypeptide 1	4011.7	2
NM_008938	***Prph2***	Peripherin 2	1448.2	367.1
NM_012065	***Pde6g***	Phosphodiesterase 6G cGMP-specific rod γ	1269.5	552.6
NM_009073	*Rom1*	Retinal outer segment membrane protein 1	948.8	229.1
NM_015745	***Rbp3***	Retinol binding protein 3	885.3	1024
NM_009118	***Sag***	S-antigen retina and pineal gland	765.4	548.7
NM_011676	***Unc119***	Unc-119 homolog	719.1	407.3
NM_024458	*Pdc*	Phosducin	689.8	302.3
NM_009038	*Rcvrn*	Recoverin	643.6	288
NM_011099	***Pkm2***	Pyruvate kinase muscle	604.7	467.9
NM_001159730	*Pdc*	Phosducin	580	250.7
NM_146079	*Guca1b*	Guanylate cyclase activator 1B	552.6	68.1
NM_008131	***Glul***	Glutamate-ammonia ligase	545	530.1
NM_001136074	*Nrl*	Neural retina leucine zipper	545	1.9
NM_001160017	*Gnb1*	Guanine nucleotide binding protein β polypeptide 1	487.8	24.4
NM_011428	***Snap25***	Synaptosomal-associated protein 25 kDa	471.1	576
NM_146086	*Pde6a*	Rod photoreceptor cGMP phosphodiesterase a subunit	433.5	35.8
NM_026358	***4930583H14Rik***	Unknown	407.3	362
NM_013415	***Atp1b2***	ATPase Na^+^K+ transporting β 2 polypeptide	369.6	608.9
NM_144921	*Atp1a3*	Α 3 subunit of Na^+^K+ ATPase	367.1	286
NM_008806	*Pde6b*	Phosphodiesterase 6B cGMP-specific rod β	364.6	16.2
NM_001160016	*Gnb1*	Guanine nucleotide binding protein β polypeptide 1	352.1	18.1
	*(isoform 2)*			
NM_008142	*Gnb1*	Guanine nucleotide binding protein β polypeptide 1	349.7	17.9
	*(isoform 1)*			
NM_010314	*Gngt1*	Guanine nucleotide binding protein g transducing activity polypeptide 1	340.1	203.7

As photoreceptors constitute almost 70% of cells in P21 WT and *Nrl^−/−^* retina, the high expressed genes (in bold) likely encode proteins associated with general photoreceptor function/metabolism. WT=wild type

**Table 7:** Top 25 highly expressed transcripts in *Nrl−/−* retina based on RNA-seq data.

**Transcript ID**	**Gene ID**	**Gene name**	**WT FPKM**	**Nrl^−/−^ FPKM**
NM_007538	*Opn1sw*	Opsin 1 short-wave-sensitive	149.1	2740.1
NM_015745	***Rbp3***	Retinol binding protein 3	885.3	1024
NM_008141	*Gnat2*	Guanine nucleotide binding protein a transducing 2	70.5	861.1
NM_013530	*Gnb3*	Guanine nucleotide binding protein β polypeptide 3	103.3	786.9
NM_133205	*Arr3*	Arrestin 3, retinal	69.6	652.6
NM_023898	*Pde6h*	Phosphodiesterase 6H cGMP-specific cone g	91.1	634.7
NM_013415	***Atp1b2***	ATPase Na^+^K+ transporting β 2 polypeptide	369.6	608.9
NM_011428	***Snap25***	Synaptosomal-associated protein 25 kDa	471.1	576
NM_012065	***Pde6g***	Phosphodiesterase 6G cGMP-specific rod g	1269.5	552.6
NM_009118	***Sag***	S-antigen retina and pineal gland	765.4	548.7
NM_008131	***Glul***	Glutamate-ammonia ligase	545	530.1
NM_053245	*Aipl1*	Aryl hydrocarbon receptor interacting protein-like 1	313	515.6
NM_009305	*Syp*	Synaptophysin	326.3	505
NM_008189	*Guca1a*	Guanylate cyclase activator 1A (retina)	306.6	487.8
NM_011099	***Pkm2***	Pyruvate kinase muscle	604.7	467.9
NM_013494	*Cpe*	Carboxypeptidase E	337.8	439.6
NM_023121	*Gngt2*	Guanine nucleotide binding protein g transducing activity polypeptide 2	32	407.3
NM_011676	***Unc119***	Unc-119 homolog	719.1	407.3
NM_021273	*Ckb*	Creatine kinase brain	290	398.9
NM_007450	*Slc25a4*	Solute carrier family 25 member 4	313	385.3
NM_008938	***Prph2***	Peripherin 2	1448.2	367.1
NM_026358	***4930583H14Rik***	Unknown	407.3	362
NM_016774	*Atp5b*	ATP synthase H^+^ transporting mitochondrial F1 complex β polypeptide	315.2	352.1
NM_001038664	*Gngt2*	Guanine nucleotide binding protein γ transducing activity polypeptide 2	22.6	337.8
NM_010106	*Eef1a1*	Eukaryotic translation elongation factor 1 α 1	265	328.6

As photoreceptors constitute almost 70% of cells in P21 WT and *Nrl^−/−^* retina, the high expressed genes (in bold) likely encode proteins associated with general photoreceptor function/metabolism. WT=wild type

### Rod and cone photoreceptor enriched genes

**Figure 5:**
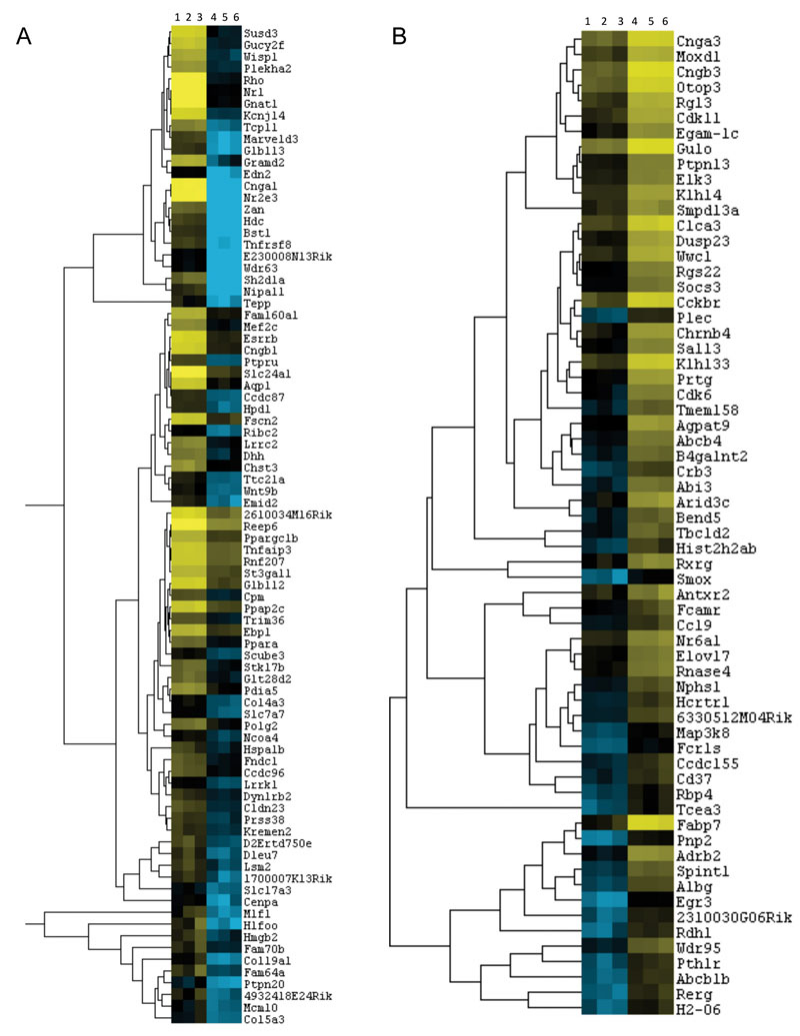
Heatmaps and hierarchical clusters of differentially expressed rod-specific genes and cone-specific genes or those involved in retinal remodeling. Heatmaps with dendrograms of clusters of differentially expressed rod genes (**A**) and cone / retinal remodeling genes (**B**) by applying hierarchical clustering. A filtered list of mRNA transcript isoforms was further revised for fold change ≥1.5 and p-value <0.05, and duplicate gene symbol rows were deleted to retain the most expressed isoform as reflective of the gene. This list was used to generate the heatmap and the master cluster. Specific clusters of rod specific genes and cone-specific or retinal remodeling genes were identified as clusters containing known rod genes (e.g., Rhodopsin [*Rho*], guanine nucleotide binding protein, alpha transducing 1 [*Gnat1*], cyclic nucleotide gated channel alpha 1 [*Cnga1*], and nuclear receptor subfamily 2, group E, member 3 [*Nr2e3*]) and cone genes (e.g., fatty acid binding protein 7, brain [*Fabp7*], cyclic nucleotide gated channel alpha 3 [*Cnga3*], cyclic nucleotide gated channel beta 3 [*Cngb3*]). Columns 1, 2, and 3 are wild-type samples, and columns 4, 5, and 6 are *Nrl^−/−^* samples.

We then focused on DEGs between the *Nrl^−/−^* and WT retinas. A total of 1,422 transcripts, corresponding to 1,218 unique genes, showed a minimum fold change of 1.5 at p≤0.05. Hierarchical clustering of all differentially expressed transcripts resulted in two distinct clusters: one cluster of 477 genes downregulated in the *Nrl^−/−^* retina includes all known rod-specific genes such as rhodopsin (*Rho*; FC=-4,804), guanine nucleotide binding protein, alpha transducing 1 (*Gnat1*; FC=-2,034), and nuclear receptor subfamily 2, group E, member 3 (*Nr2e3*; FC=-227.5; Figure 5A and Table 8); and the other cluster of 741 upregulated genes had all cone-specific genes such as opsin 1, short-wave-sensitive (*Opn1sw*; FC=18.4), cyclic nucleotide gated channel beta 3 (*Cngb3*; FC=18.1), and *Gnat2* (FC=12.2; Figure 5B and Table 9).

**Table 8:** Top 25 transcripts down-regulated in *Nrl^−/−^* retina compared to WT.

**Transcript**	**Gene ID**	**Gene name**	**WT FPKM**	**Nrl^−/−^ FPKM**	**FC**
NM_145383	Rho	Rhodopsin	8135.4	1.7	−4803.93
NM_008140	Gnat1	Guanine nucleotide binding protein γ transducing activity polypeptide 1	4011.7	2	−2033.85
NM_001136074	Nrl	Neural retina leucine zipper	545	1.9	−284.05
NM_007723	Cnga1	Intracellular cGMP activated cation channel	232.3	1	−229.13
NM_013708	Nr2e3	Nuclear receptor subfamily 2 group E member 3	237.2	1	−227.54
NM_144813	Slc24a1	Solute carrier family 24 member 1 (sodium-potassium-calcium exchanger 1)	263.2	3.2	−81.57
NM_145963	Kcnj14	Potassium inwardly-rectifying channel subfamily J member 14	63.6	1.4	−44.02
NM_139292	Reep6	Receptor accessory protein 6	317.4	8.6	−36.76
NM_025491	Susd3	Sushi domain containing 3	58.1	1.7	−34.06
NM_011934	Esrrb (isoform 1)	Estrogen-related receptor β	67.2	2.6	−26.17
NM_001007576	Gucy2f	Guanylate cyclase 2f	36.5	1.6	−22.78
NM_008806	Pde6b	Phosphodiesterase 6B cGMP-specific rod β	364.6	16.2	−22.32
NM_001195413	Cngb1	Cyclic nucleotide gated channel β 1	52.3	2.5	−21.11
NM_001160017	Gnb1 (isoform 3)	Guanine nucleotide binding protein β polypeptide 1	487.8	24.4	−19.97
NM_008736	Nrl	Neural retina leucine zipper	20	1	−19.97
NM_008142	Gnb1 (isoform 1)	Guanine nucleotide binding protein β polypeptide 1	349.7	17.9	−19.7
NM_007472	Aqp1	Aquaporin 1	42.5	2.2	−19.56
NM_001160016	Gnb1 (isoform 2)	Guanine nucleotide binding protein β polypeptide 1	352.1	18.1	−19.29
NM_153803	Glb1l2	Galactosidase, β 1-like 2	55.7	3.5	−16.11
NM_001159500	Esrrb (isoform 2)	Estrogen-related receptor β	22.5	1.5	−15.03
NM_172802	Fscn2	Fascin homolog 2, actin-bundling protein, retinal	41.6	3	−14.03
NM_001033498	Gramd2	Member of the GRAM domain containing family	20.3	1.4	−14.03
NM_027001	2610034M16Rik	Unknown	69.6	5.1	−13.64
NM_018865	Wisp1	WNT1 inducible signaling pathway protein	17.8	1.4	−12.21
NM_146086	Pde6a	Rod photoreceptor cGMP phosphodiesterase α subunit	433.5	35.8	−12.13

Many of the genes down-regulated in *Nrl^−/−^* retina represent rod-specific transcripts, whereas upregulated genes include those associated with cone function and retinal remodeling.

**Table 9:** Top 25 transcripts upregulated in *Nrl^−/−^* retina compared to WT.

**Transcript**	**Gene ID**	**Gene title**	**WT FPKM**	**Nrl^−/−^ FPKM**	**FC**
NM_021272	Fabp7	Fatty acid binding protein 7 brain	2.5	62.7	24.59
NM_007538	Opn1sw	Opsin 1 short-wave-sensitive	149.1	2740.1	18.38
NM_013927	Cngb3	Cyclic nucleotide gated channel β 3	4.7	85.6	18.13
NM_033614	Pde6c (isoform 1)	Phosphodiesterase 6C, cGMP-specific, cone, α prime	12.6	199.5	15.78
NM_001170959	Pde6c (isoform 2)	Phosphodiesterase 6C, cGMP-specific, cone, α prime	13.8	207.9	14.93
NM_001038664	Gngt2 (isoform 2)	Guanine nucleotide binding protein γ transducing activity polypeptide 2	22.6	337.8	14.93
NM_001166651	Klhl33	Kelch-like 33	3	42.2	14.03
NM_007627	Cckbr	Cholecystokinin B receptor	3.6	50.2	13.83
NM_017474	Clca3	Chloride channel calcium activated 3	3.4	46.2	13.64
NM_027132	Otop3	Otopetrin 1 homolog	4.4	56.5	12.82
NM_023121	Gngt2 (isoform 1)	Guanine nucleotide binding protein γ transducing activity polypeptide 2	32	407.3	12.73
NM_178747	Gulo	L gulonolactone oxidase	6.5	82.7	12.64
NM_008141	Gnat2	Guanine nucleotide binding protein γ transducing activity polypeptide 2	70.5	861.1	12.21
NM_145574	Ccdc136	Coiled-coil domain containing 136	17.6	213.8	12.13
NM_009918	Cnga3	Cyclic nucleotide gated channel α 3	5	54.6	11
NM_133205	Arr3	Arrestin 3, retinal	69.6	652.6	9.32
NM_184053	Calu	Calumenin, a calcium ion binding protein	14.8	131.6	8.82
NM_183224	Fam19a3	Family with sequence similarity 19, member A3	30.7	266.9	8.69
NM_010164	Eya1	Eyes absent 1 homolog	1.4	12.6	8.63
NM_001201378	Ccdc136	Coiled-coil domain containing 136	16.6	142	8.51
NM_025659	Abi3	ABI gene family, member 3	1.4	11.5	8.28
NM_013848	Ermap	Erythroblast membrane-associated protein	1.2	9.8	8
NM_133167	Parvb	Parvin β	9	71.5	7.94
NM_007419	Adrb1	Β-1 adrenergic receptor	6.5	50.2	7.73
NM_138653	Bspry	B-box and SPRY-domain containing (zetin-1)	1.4	10.6	7.73

Many of the genes down-regulated in *Nrl^−/−^* retina represent rod-specific transcripts, whereas upregulated genes include those associated with cone function and retinal remodeling.

**Figure 6:**
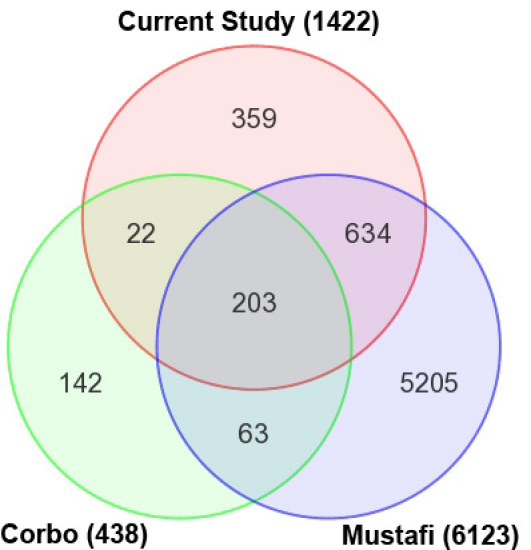
Comparison of the current and previous data sets of differentially expressed transcripts of *Nrl^−/−^* versus wild type (WT) mouse retina. Overlap between the differentially expressed transcripts (DETs) identified in the current study and previous studies using mouse retinas from the same age and genotype was determined using the Mouse Genome Informatics (MGI) gene symbol as the identifier. The current study includes all mRNA transcripts identified with the Burrows-Wheeler Aligner (BWA) workflow (fold change ≥1.5 and p value <0.05). The 438 DETs of an Affymetrix microarray study [38] and 6,123 DETs from another RNA-seq study [54] with similar criteria were used for comparison with our study. Of the 438 DETs from the Corbo et al. [38] study, 157 transcripts are not significantly differentially expressed in our data, 11 are expressed below the fragments per kilobase of exon model per million mapped reads (FPKM) detection threshold of 1.0, and 38 do not map to the current annotations. Of the 6,123 DETs from the Mustafi et al. [54] study, 4,858 transcripts are not significantly differentially expressed in our study, 348 are expressed below our FPKM detection threshold of 1.0, and 62 do not map to the current annotations.

We then compared our DEG list with two published studies that examined WT and *Nrl^−/−^* retinas: a recent transcript-level RNA-seq analysis that included 6,123 DETs [54] and a gene-level microarray analysis showing 438 DEGs [38] (Figure 6). To obtain the list of DEGs from the Mustafi et al. [55] data set, we performed ANOVA on their FPKM data from GEO database. Interestingly, the DEGs lists from the three studies had only 203 common genes including many previously identified genes specifically expressed in cone (fatty acid binding protein 7, brain [*Fabp7*], *Opn1sw*, *Cngb3*, and *Gnat2*) or rod (*Rho*, *Gnat1*, cyclic nucleotide gated channel alpha 1 [*Cnga1*], and *Nr2e3*) photoreceptors. To assess the power of RNA-seq to more comprehensively identify DETs than microarray, we examined the list of 634 genes identified in common by the RNA-seq studies but not by the microarray study. This list included 18 retinal disease genes (ATP-binding cassette, sub-family A (ABC1), member 4 [*Abca4*], cadherin 23 (otocadherin) [*Cdh23*], ADP-ribosylation factor-like 6 [*Arl6*], Bardet-Biedl syndrome 9 (human) [*Bbs9*], calcium binding protein 4 [*Cabp4*], cyclic nucleotide gated channel alpha 3 [*Cnga3*], G protein-coupled receptor 98 [*Gpr98*], guanylate cyclase activator 1a (retina) [*Guca1a*], opsin 1 (cone pigments), medium-wave-sensitive (color blindness, deutan) [*Opn1mw*], orthodenticle homolog 2 (Drosophila) [*Otx2*], phosphodiesterase 6G, cGMP-specific, rod, gamma [*Pde6g*], peripherin 2 [*Prph2*], retinol binding protein 4, plasma [*Rbp4*], retinol dehydrogenase 1 (all trans) [*Rdh1*], regulator of G-protein signaling 9 binding protein [*Rgs9bp*], unc-119 homolog (C. elegans) [*Unc119*], Usher syndrome 2A (autosomal recessive, mild) homolog (human) [*Ush2a*], and whirlin [*Whrn*]) and several known genes involved in visual perception (guanylate cyclase 2e [*Gucy2e*], guanylate cyclase 2f [*Gucy2f*], recoverin [*Rcvrn*], RAR-related orphan receptor beta [*Rorb*], and sal-like 3 (Drosophila) [*Sall3*]). Several genes showing large differential expression values might participate in rod homeostasis (galactosidase, beta 1-like 2 [*Glb1l2*] FC=-14.02, GRAM domain containing 2 [*Gramd2*] FC=-14.0, carbohydrate (chondroitin 6/keratan) sulfotransferase 3 [*Chst3*] FC=-4.8, desert hedgehog [*Dhh*] FC=-4.1, and ADP-ribosylation factor-like 4D [*Arl4d*] FC=-3.6) and cone function (dual specificity phosphatase 23 [*Dusp23*] FC=6.3, cyclin-dependent kinase 11B [*Cdkl1*] FC=6.1, tryptophan hydroxylase 1 [*Tph1*] FC=4.7, muscle glycogen phosphorylase [*Pygm*] FC=4.6, cyclin-dependent kinase 6 [*Cdk6*] FC=4.0, *Sall3* FC=3.9, and early growth response 1 [*Egr1*] FC=3.7).

Our RNA-seq data allowed us to identify 359 genes not identified in previous investigations. To further assess the quality of our analysis, we performed qRT–PCR validation of 15 genes identified by other studies (but not in our study) as differentially expressed and of 7 genes uniquely identified by our study (but not by other studies; Table 10). Of the 15 genes identified by other studies, only three (ATP-binding cassette, sub-family A (ABC1), member 13 [*Abca13*], CD8 antigen, alpha chain [*Cd8a*], and acyl-CoA oxidase-like [*Acoxl*]) were confirmed with qRT–PCR as being differentially expressed. We also detected these three as differentially expressed but had filtered them out because of FPKM values that were less than 1.0 in all samples. Interestingly, the *Abca13* transcript detected in the retina had only sequence reads for exons 56 through 62. This finding was supported by qRT–PCR using two SYBR assays designed to exons 53/55 and exons 56/58. All seven genes uniquely identified by our study were validated as significantly differentially expressed.

**Table 10:** Validation of selected genes from study comparisons by qRT–PCR

**Name**	**Transcript ID**	**qRT–PCR FC**	**Corbo FC**	**Mustafi FC**	**This report FC**	**p-value**	**WT FPKM**	**Nrl^−/−^ FPKM**
**Corbo**								
Abca13 exon 53/55	NM_178259	NA	−11.58	NA	−4.18	0.000278	0.75	0.18
Abca13 exon 56/58	NM_178259	−21.36	−11.58	NA	−4.18	0.000278	0.75	0.18
Amz2	NM_025275	1.09	−6.77	1.13	1.06	0.1122	15.06	16.13
Klf9	NM_010638	1.09	−2.6	−1.15	1.11	0.2481	29.7	33.12
Pip5k1a	NM_008847	1.43	50	1.03	1.35	0.0015	11.81	16.25
Sema7a	NM_011352	1.03	20	−1.39	1.13	0.0634	13.33	15.19
Txnip	NM_023719	−1.07	12.5	1	1.04	0.7347	2.17	2.01
**Mustafi**								
Cox5b	NM_009942	1.26	NA	−12.15	1.01	0.9342	18.31	18.24
Drd4	NM_007878	−1.23	NA	−8.72	−1.92	0.0764	39.6	20.92
Cd8a	NM_009857	11.5	NA	21.17	9.03	3.47E-06	0.08	0.73
Ctss	NM_021281	1.25	NA	6.92	1.29	0.0228	3.82	5.23
**Corbo-Mustafi**								
Acoxl	NM_028765	−28.6	−13.93	−34	−4.61	0.0291	0.28	0.06
Rpgrip1	NM_001168515	−1.3	−2.32	−1.83	1.06	0.1157	29.94	31.85
Dynlt3	NM_025975	1.28	5	3.45	1.46	0.0036	17.79	26.39
Rab18	NM_181070	1.37	3.23	3.31	1.29	0.0028	33.71	43.77
Neurod1	NM_010894	1.38	2.63	2.2	1.14	0.0888	168.51	192.86
**This report**								
Plekhf2	NM_175175	−5.88	NA	−1.35	−5.35	0.000428	37.77	7.21
Klhl3	NM_001195075	−6.9	NA	NA	−3.29	0.000101	8.62	2.6
Ccdc24	NM_001034876	−2.93	NA	NA	−2.64	0.0031	66.86	24.85
Rgs22	NM_001195748	11.24	NA	NA	3.84	5.93E-07	1.95	7.47
Hr	NM_021877	3.82	NA	1.25	3.89	0.000307	16.38	63.51
Wscd2	NM_177292	4.1	NA	1.13	4	0.000123	5.73	22.91
Klhl33	NM_001166651	27.12	NA	NA	14.03	2.34E-06	3.02	42.25

Differential expression of genes identified in three global profiling studies (Corbo, Mustafi and this report) was validated by qRT–PCR. Genes shown under the Corbo and Mustafi subheadings were identified in respective studies, but not in the current study. Corbo-Mustafi subheading indicates genes identified in both Corbo and Mustafi studies but not in the current report. The two genes, *Cd8a* and *Acoxl,* identified previously were differentially expressed by qRT–PCR but were filtered out of our sequencing data because of the low FPKM values observed. The gene *Abca13* was detected as only expressing the last 7 exons of the annotated sequence. qRT–PCR assays that distinguished between the two isoforms were able to confirm the differential expression of the last 7 *Abca13* exons. This gene was filtered out of our sequencing results because of the low FPKM value observed. All genes included in this report subheading were uniquely identified by the current study (and not in Corbo and Mustafi reports). FC, p-val, and FPKM values in this report are derived from the sequencing results reported here. Genes were selected as having the largest fold changes in each category and from current annotation in the latest mm9 Refseq build. All differentially expressed genes identified in this report could be validated by qRT–PCR. FC=fold change, NA=not applicable, FPKM=fragments per kilobase exon model per million reads.

The significantly lower number of DETs detected by our study compared to the Mustafi et al. study (2011; 1,422 versus 6,123, respectively) can be attributed to the following:

1. We used a stringent 1.0 FPKM cutoff that generated a list of genes with significant base level expression and fewer false positives than a lower expression level threshold. If we had decreased our threshold to 0.1 FPKM, we would have detected 975 more DETs; however, these genes are expressed at an extremely low level and their impact must be weighed against the increase in false positives. We chose a conservative criterion to identify significant and bona fide differentially expressed genes.

2. Mustafi et al. [54] pooled multiple RNA samples before generating the library and used the identical library on multiple lanes of the sequencer. Our experimental design consisted of libraries generated from individual biologic replicates that allowed us to eliminate the transcripts based on p-value.

Several DETs we identified might contribute to photoreceptor function but are not yet characterized; these include pleckstrin homology domain containing, family F (with FYVE domain) member 2 (*Plekhf2*; FC=-5.35), kelch-like 13 (Drosophila) [*Klhl3*] (FC=-3.3), NIPA-like domain containing 1 (*Nipal1*; FC=-2.8), and coiled-coil domain containing 24 (*Ccdc24*; FC=-2.6) in the WT retina, and kelch-like 33 (Drosophila) [*Klhl33*] (FC=14), WSC domain containing 2 (*Wscd2*; FC=4), hairless (*Hr*; FC=3.9) and regulator of G-protein signaling 22 (*Rgs22*; FC=3.8) in the *Nrl^−/−^* retina. We also identified Crx opposite strand transcript 1 (*Crxos1*; FC=4.1), which is exclusively expressed in the eye from the opposite strand of a key retinal transcription factor, cone-rod homeobox containing gene (*Crx*) [55]. An interesting new finding is the retinal expression of multiple genes from the Kelch-like family (Klhl3, 4, 5, 18, 33, 36), solute carrier family (>30 members), and zinc-finger protein family (>10 members). Mutations in at least one gene from each family have previously been associated with retinal disease: Klhl7 with autosomal dominant RP [56], Slc24A1 with autosomal-recessive congenital stationary night blindness [57], and Znf513 with autosomal-recessive retinitis pigmentosa (RP) [58].

## Discussion

Specific patterns of gene expression define the morphology and function of distinct cell types and tissues. Changes in gene expression are associated with complex biologic processes, including development, aging, and disease pathogenesis. Until recently, such investigations focused on one or a few genes at a time. Advances in genomic technology have permitted simultaneous evaluation of most, if not all, genes that respond to an extrinsic microenvironment or intrinsic biologic program(s). Such studies are critical for delineating gene networks that can be targeted for treating specific diseases. RNA-seq allows comprehensive evaluation of transcriptomes, alternative transcripts, and coding polymorphisms. However, analyzing RNA-seq data has been challenging due to the complexity associated with quality control, sequence alignments, and handling of large data sets [59]. Several algorithms [45,60] have been proposed for mapping sequence reads to the reference genome, and multiple workflows [16,50] suggested for RNA-seq data analysis. Here, we report a detailed RNA-seq methodology using WT and *Nrl^−/−^* retinas as a study paradigm and establish the high performance of NGS technology compared to microarray and qRT–PCR platforms for transcript identification and quantification studies. Consistent with recent studies [61], our RNA-seq data demonstrate high sensitivity, a wider dynamic range of coverage, and lower technical variability.

Quantitative RT–PCR has long been considered the “gold standard” for mRNA quantification [62,63], and hence routinely used to validate results from transcriptome analysis studies. We show that FPKM values from RNA-seq analysis have a strong linear correlation across at least four orders of magnitude with Ct values from qRT–PCR. Expression of several HKGs is underestimated by RNA-seq because of the algorithmic limitation associated with alignment of reads that map to multiple genomic locations (paralogous sequences or pseudogenes). All of the outlying HKGs inspected had a lower-than-projected FPKM value due to varying numbers of associated pseudogenes [64-67]. For example, *Gapdh* has 331 pseudogenes in the mouse genome [64]. Our qRT–PCR data projected an FPKM value of approximately 4000 for *Gapdh*, yet the BWA workflow estimated an FPKM of only 6.6 in the WT retina (see Figure 3). This was also the case, but less severe, for *Pgk1*, *Rps26*, *Rpl13a*, and *ActB*. Current algorithms proportionally divide the number of reads aligning to multiple genes during FPKM calculation among those genes. In our study, unsuitable qRT–PCR assay design explains the remaining exceptions to the linear correlation between qRT–PCR and RNA-seq. After careful visual inspection of the aligned reads in IGV, we found that the assay designed for *Wisp1* was not specific to the splice variant expressed in the retina. Similarly, the assays designed for *Cadm3* and *Ubc* were specific to one of the two transcripts expressed in the retina. Hence, RNA-seq provides a better assessment of alternate isoforms, and transcript quantification is not limited by the design of qRT–PCR assays.

We took advantage of the RNA-seq data to inspect the expression of commonly used HKGs (see Table 5) for normalization in qRT–PCR assays. The choice generally depends on specific tissue and/or developmental time points being investigated. Our RNA-seq studies suggest that most of the HKGs can be used for normalization calculation in qRT–PCR assays; however, *Gapdh*, β-2 microglobulin (*B2m*), and *Ubc* do not appear to be good choices. Additional RNA-seq data would help in delineating relevant HKGs appropriate for qPCR validation in developing retina or cell types.

We compared two different strategies for analyzing WT and *Nrl^−/−^* RNA-seq data. The BWA workflow relies on fast and accurate gapped alignment of reads to the exonic regions of the genome. Since the gap between most adjacent exons is larger than a few bases, the cumulative gap extension penalty underestimates the quality of the alignment of reads spanning the splice junctions. Hence, the BWA workflow produced accurate quantitative estimation of gene and transcript isoform expression while losing valuable information about alternate splicing. The higher accuracy of quantitative gene expression estimates by the BWA workflow compared to those by TopHat is evident from the stronger correlation determined by linear regression analysis of the DETs. The regression line from BWA had a slope of −1.056 (compared to −0.905 for TopHat) and R^2^ of 0.8798 (compared to 0.7727 for TopHat).

The TopHat workflow maps the reads to exonic regions of the reference genome as well as across all known and putative splice junctions defined in the Ensembl GTF file. TopHat attempts to map reads across splice junctions defined in the Ensembl GTF file and across novel splice junctions detected during the first phase of alignment. Hence, the TopHat workflow maps significantly more reads starting with the same number of pass filter (PF) reads and detects additional transcript isoforms missed by the BWA workflow. The source of genomic annotations used by these methods is another important difference. UCSC refFlat annotation (used by the BWA workflow) for the mouse reference genome (build mm9) contained approximately 28,000 unique transcript isoforms, whereas the Ensembl GTF file (used by the TopHat workflow) for the same genome build listed three times more unique transcript isoforms. The problem is amplified because of the lack of one-to-one mapping for several transcripts defined in the UCSC refFlat file in Ensembl GTF. Hence, a non-trivial number of DETs detected by the BWA workflow could not be mapped to any DET from the TopHat workflow (see Figure 2, regions shaded in green).

The BWA workflow detects about 16,000 transcripts in the retina, with a minimum expression equivalent to one transcript per cell (i.e., 1 FPKM) [16]. When the criteria were relaxed to cover transcripts expressed at low levels (0.1 FPKM), 20,707 transcripts were detected in the retina. This is not surprising as the whole retina includes more than 50 distinct neuronal cell types, and each cell would achieve protein diversity largely by alternative promoter usage and/or alternative splicing [68]. The TopHat workflow yields thousands of known and putative transcript isoforms not previously described in the retina. However, validating these novel isoforms predicted from RNA-seq data remains a challenge.

Integrated analysis of RNA-seq data with miRNA-seq, transcription factor binding sites data (chromatin immunoprecipitation sequencing-Chip-Seq), genetic variations (expression Quantitative Trait Loci) [69], and methylation patterns would allow decoding of the complex regulatory networks associated with retinal development and function. Several technical improvements would however be necessary to overcome the bias introduced into the RNA-seq data due to GC content, mappability of reads, length of the gene, and regional differences due to local sequence structure [70]. RNA-seq methods are more likely to identify longer differentially expressed transcripts than shorter transcripts with the same effect size [71]. New statistical methods are being developed to correct for systematic biases inherent in NGS data [70-72]. In the coming years, we will witness an explosion in high throughput genomic methods that are expected to revolutionize biology and biomedical discovery.
